# Evaluating ChatGPT text mining of clinical records for companion animal obesity monitoring

**DOI:** 10.1002/vetr.3669

**Published:** 2023-12-06

**Authors:** Ivo S. Fins, Heather Davies, Sean Farrell, Jose R. Torres, Gina Pinchbeck, Alan D. Radford, Peter‐John Noble

**Affiliations:** ^1^ Small Animal Veterinary Surveillance Network Institute of Infection Veterinary and Ecological Sciences University of Liverpool Liverpool UK; ^2^ Department of Computer Science Durham University Durham UK; ^3^ Institute for Animal Health and Food Safety University of Las Palmas de Gran Canaria Las Palmas, Gran Canaria Spain

## Abstract

**Background:**

Veterinary clinical narratives remain a largely untapped resource for addressing complex diseases. Here we compare the ability of a large language model (ChatGPT) and a previously developed regular expression (RegexT) to identify overweight body condition scores (BCS) in veterinary narratives pertaining to companion animals.

**Methods:**

BCS values were extracted from 4415 anonymised clinical narratives using either RegexT or by appending the narrative to a prompt sent to ChatGPT, prompting the model to return the BCS information. Data were manually reviewed for comparison.

**Results:**

The precision of RegexT was higher (100%, 95% confidence interval [CI] 94.81%–100%) than that of ChatGPT (89.3%, 95% CI 82.75%–93.64%). However, the recall of ChatGPT (100%, 95% CI 96.18%–100%) was considerably higher than that of RegexT (72.6%, 95% CI 63.92%–79.94%).

**Limitations:**

Prior anonymisation and subtle prompt engineering are needed to improve ChatGPT output.

**Conclusions:**

Large language models create diverse opportunities and, while complex, present an intuitive interface to information. However, they require careful implementation to avoid unpredictable errors.

## INTRODUCTION

Obesity is a common and significant medical condition in companion animals.^1^, ^2^ The Small Animal Veterinary Surveillance Network (SAVSNET) collects anonymised electronic health records (EHRs) from veterinary practices in real time.^3^ These remain an unexploited resource for investigating canine health, with relevant clinical information often submerged in unstructured free text. Automated systems to surface this information are therefore essential where reading and manual annotation is not feasible. Regular expressions, tools designed to detect fixed word patterns, have often been used in this setting. Such methods try to identify implicit negation (‘not vomiting’) and contextual information that indicates a feature is not present (‘come back if there are any signs of vomiting’) and require complex rules to accommodate varied/unpredictable language.^4^

Recently, large language models (LLM), including generative pre‐trained transformers (such as GPT3.5, which underpins ChatGPT) have become available. These complex neural networks, with hundreds of billions of parameters,^5^ trained using vast datasets to generate responses to preceding text^4^, ^5^, ^6^ can generate human‐like responses to complex prompts. These provide an exciting opportunity for automated data extraction,^7^, ^8^, ^9^ and studies assessing their veterinary application are urgently required. In human healthcare research and practice, identified benefits of the application of LLM include efficient analysis of healthcare datasets, streamlining practice workflows and a step ahead towards personalised medicine.^10^ Nonetheless, highlighted risks include inaccurate content associated with the risk of hallucinations, the risk of bias and cybersecurity issues, with some authors highlighting the need for regulators and healthcare professionals to be engaged in this paradigm shift on how patients access information.^10^, ^11^

Here, using a sample of publicly available clinical narratives, we compare the performance of a validated rule‐based system using regular expressions (RegexT^12^) to that of a prompt‐based approach using ChatGPT to identify the body condition score (BCS) of a patient, if recorded at the time of the consultation.

## MATERIALS AND METHODS

SAVSNET collects EHRs from a sentinel network of UK veterinary practices. Each EHR contains a clinical narrative written by the attending practitioner. This study used a random sample of 4415 pre‐anonymised, publicly available companion animal EHRs.^13^ These narratives were read by domain experts to identify overweight BCSs recorded either on a five‐point scale (≥3.5 out of 5 are considered overweight) or a nine‐point scale (≥6 out of 9 are considered overweight). The collection and use of EHRs by SAVSNET is approved by the Research Ethics Committee at the University of Liverpool (RETH001081).

**FIGURE 1:**
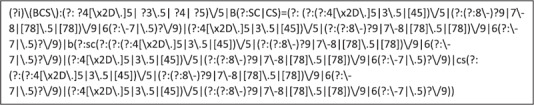
Regular expression used to identify overweight body condition scores

A regular expression (RegexT) was designed to detect overweight BCSs considering the variety of notations used by practitioners to record both the denominator and numerator in free text (Figure 1).

**FIGURE 2:**
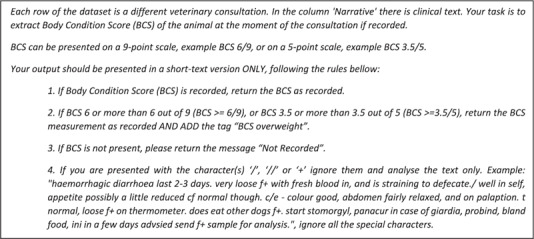
ChatGPT prompt used to generate an output reporting body condition score (BCS) and overweight status

In parallel, we refined a ChatGPT prompt describing four basic rules for an output of any BCS along with a prediction regarding overweight status. Data extraction was performed using Python (version 3.7.10).^14^ GPT3.5 Turbo^15^ was accessed through a Python application programming interface processing multiple EHRs, each a separate row, appended to the prompt in Figure 2.

The accuracy of these RegexT and ChatGPTs systems was assessed based on the returned BCS using precision (equivalent to positive predictive value) and recall (equivalent to sensitivity). The 95% confidence intervals (95% CI) were calculated with the modified Wald method using GraphPad (QuickCalcs).^16^

## RESULTS

Manual reading of the 4415 records identified 117 (2.65%) reporting an overweight BCS.

ChatGPT identified all 117 overweight BCSs (recall 100%, 95% CI 96.18%–100%). The output variably fitted the structure requested by the prompt, requiring some manual interpretation. Only 89 were additionally described as being overweight. Twelve GPT outputs appended text either peripheral to the task or generally not relevant to weight, such as ‘Last WORM and FLEA treatments?’ and ‘Possible dietary indiscretion’; however, some of these additional texts were relevant (‘Coming down in weight. 600 g since last September. O thinks because he is getting less treats’.).

ChatGPT falsely identified BCSs in 14 records; these reflected extraction of other similarly formatted clinical information such as lameness scoring (e.g., EHR‐text ‘6/10 lameness’; ChatGPT‐output ‘BCS 6/9, BCS overweight’) and body weight information (e.g., EHR‐text ‘wt 6.65 kg’; ChatGPT‐output ‘BCS 6.65/9’). The precision of ChatGPT was therefore 117 of 131 (89.3%, 95% CI 82.75%–93.64%).

ChatGPT classified an additional 61 consultations as being overweight in the absence of a recorded overweight BCS. Forty of these were also described as overweight by the attending veterinarian (e.g., ‘she is overweight’, ‘normal on clinical exam apart from overweight’). Of the remaining 21 records, ChatGPT recorded a normal or low BCS as overweight or an erroneous false‐positive high BCS. For example, ChatGPT coded ‘BCS 5.75/9 would benefit from further weight loss’ as overweight despite BCS not crossing the threshold of 6.

ChatGPT failed to return an appropriate answer for 29 records, instead outputting texts such as ‘Hello! How can I assist you today?’. This was often associated with short narratives (27 were less than 23 characters in length), such as ‘pay now’ and ‘all ok sign off’, none containing BCS information.

In comparison, from the same 4415 records, RegexT successfully identified 85 of the 117 (recall 72.6%, 95% CI 63.92%–79.94%) narratives containing an overweight BCS and returned no false positives (precision 100%, 95% CI 94.81%–100%). The 32 overweight BCSs missed were associated with format variants not captured by the regex syntax (e.g., ‘BCS: 6–7 out of 9’ and ‘BCS: 6/9’). Clearly, the regex did not identify any of the 40 narratives that lacked a BCS but that were described as overweight by the veterinarian and identified by ChatGPT.

A full list of the narratives and the regex and ChatGPT outputs is available in Supporting Information.

## DISCUSSION

To leverage the true value of clinical free text in large health datasets to understand complex diseases such as obesity will require the careful application of increasingly complex text mining solutions. Regular expressions have been used to identify a wide range of disease phenotypes based on coded patterns of text. More recently, LLMs have offered novel solutions in a wide range of situations. Here, we assessed the strengths and weaknesses of an LLM (GPT 3.5 Turbo) for a named entity recognition task identifying overweight BCSs in clinical veterinary EHRs, comparing the results to a regular expression and to manual reading.

A refined and contextualised ChatGPT prompt was developed to identify both all overweight BCSs and overweight animals without a reported BCS. Prompt engineering included providing ChatGPT with a clinical narrative example, as presented in point 4 of the final prompt (Figure 2), to mitigate cases where ChatGPT failed to produce useable output due to the presence of special characters in the text. For performance comparison with the previously developed RegexT and manual annotation, we only considered narratives where an overweight BCS assessment was recorded. In comparison with ChatGPT performance, the regex method missed BCSs with novel unpredicted formatting. In this setting of obesity, ChatGPT successfully identified most overweight animals, with or without a BCS and could be further used to aid the reengineering of systems based on regular expressions. However, occasional false positives were identified by ChatGPT, often associated with other scores such as lameness; this behaviour might be avoidable through more subtle prompt engineering (e.g., by prompting: ‘exclude lameness scores normally recorded out of 10 and heart murmur scores, normally recorded out of six’). When high accuracy is the goal, many case studies may still require a final manual classification. Any follow‐on manual reading task is made simpler by designing prompts that lead to tabular output from ChatGPT. However, in our study, this frequently failed, making the manual classifying step somewhat more complex than envisaged. Future studies may focus on engineering these prompts to reduce false positives and to tighten the output structure.^17^

ChatGPT could create overtly false assertions (sometimes described as hallucinations), sometimes comprising understandable unhelpful output. In other settings, these can be far more fanciful.^18^, ^19^

## CONCLUSIONS

Despite the complexity of both clinical narratives and the underlying technology of ChatGPT, we were able to successfully extract overweight BCSs with higher recall than a rule‐based system. Challenges remain around the ethics of submitting health texts to an online server; here we used a publicly available, anonymised dataset. Issues of cost prohibiting the screening of bigger datasets may be overcome by installing in‐house increasingly available free models. The two systems (regex and language model) offer possible complementarity. Future improved prompt engineering will enhance precision and outputted text format.

## AUTHOR CONTRIBUTIONS

*Hypothesis generation and experimental design*: Ivo S. Fins, Sean Farrell and Peter‐John Noble. *Data analysis and validation*: Ivo S. Fins, Heather Davies, Sean Farrell, Gina Pinchbeck, Jose R. Torres, Alan Radford and Peter‐John Noble. *Writing and revising the manuscript*: Ivo S. Fins, Alan Radford, Heather Davies, Sean Farrell and Peter‐John Noble.

## CONFLICT OF INTEREST STATEMENT

The authors declare they have no conflicts of interest.

## ETHICS STATEMENT

The collection and use of EHRs by SAVSNET is approved by the Research Ethics Committee at the University of Liverpool (RETH001081).

## Supporting information

A full list of narratives, regex and ChatGPT outputs

## Data Availability

The data that support the findings of this study are available in the supplementary material of this article.
